# Infection of *Medicago truncatula* by the Root-Knot Nematode *Meloidogyne javanica* Does Not Require Early Nodulation Genes

**DOI:** 10.3389/fpls.2020.01050

**Published:** 2020-07-09

**Authors:** Sofia R. Costa, Sabrina Chin, Ulrike Mathesius

**Affiliations:** ^1^ Division of Plant Sciences, Research School of Biology, Australian National University, Canberra, ACT, Australia; ^2^ CBMA—Centre of Molecular and Environmental Biology, Department of Biology, University of Minho, Braga, Portugal

**Keywords:** Autoregulation, abscisic acid, cytokinin, ethylene, nodulation signalling, rhizobia, root gall, root knot nematode

## Abstract

Because of the developmental similarities between root nodules induced by symbiotic rhizobia and root galls formed by parasitic nematodes, we investigated the involvement of nodulation genes in the infection of *Medicago truncatula* by the root knot nematode (RKN), *Meloidogyne javanica*. We found that gall formation, including giant cell formation, pericycle and cortical cell division, as well as egg laying, occurred successfully in the non-nodulating mutants *nfp1* (*nod factor perception1*), *nin1* (*nodule inception1*) and *nsp2* (*nodulation signaling pathway2*) and the cytokinin perception mutant *cre1* (*cytokinin receptor1*). Gall and egg formation were significantly reduced in the ethylene insensitive, hypernodulating mutant *skl* (*sickle*), and to a lesser extent, in the low nodulation, abscisic acid insensitive mutant *latd/nip* (*lateral root-organ defective/numerous infections and polyphenolics*). Despite its supernodulation phenotype, the *sunn4* (*super numeric nodules4*) mutant, which has lost the ability to autoregulate nodule numbers, did not form excessive numbers of galls. Co-inoculation of roots with nematodes and rhizobia significantly reduced nodule numbers compared to rhizobia-only inoculated roots, but only in the hypernodulation mutant *skl*. Thus, this effect is likely to be influenced by ethylene signaling, but is not likely explained by resource competition between galls and nodules. Co-inoculation with rhizobia also reduced gall numbers compared to nematode-only infected roots, but only in the wild type. Therefore, the protective effect of rhizobia on nematode infection does not clearly depend on nodule number or on Nod factor signaling. Our study demonstrates that early nodulation genes that are essential for successful nodule development are not necessary for nematode-induced gall formation, that gall formation is not under autoregulation of nodulation control, and that ethylene signaling plays a positive role in successful RKN parasitism in *M. truncatula*.

## Introduction

Plant parasitic nematodes are destructive parasites of most crop plants, estimated to cause annual crop losses of more than $1 billion world-wide (Nicol et al., 2011). This is due to the lack of effective and non-toxic control methods, but also because plant parasitic nematodes often cause unspecific above-ground symptoms that are recognized too late after infection (Moens et al., 2009; Nicol et al., 2011). Sedentary endoparasitic nematodes are thought to be the most destructive parasites as they require a living host and initiate the formation of a feeding site inside the host root, in which the female develops and consumes host resources until it lays numerous eggs that later hatch to infect new plants (Jones et al., 2013).

Root knot nematodes (RKNs) are one kind of sedentary endoparasite that cause world-wide crop losses and target hundreds of crop species. The agriculturally most relevant species include *Meloidogyne incognita*, *M. hapla*, *M. javanica, M. arenaria,* and *M. graminicola* (Moens et al., 2009; Atkinson et al., 2012). The reason for their relative lack of host specificity is unknown, but is at least partly due to the multitude of nematode effectors the nematodes inject into the host plant to produce a feeding site (Williamson and Gleason, 2003; Bird, 2004). RKNs hatch from eggs laid in a gelatinous egg mass on the root surface by mature female nematodes. The juveniles rapidly hatch and develop into the infective second stage juveniles (J2). J2s are attracted to host roots, although it is not clear which root exudates are the essential chemoattractants. Some root exudates and volatiles, including phenolic acids and flavonoids, have been shown to alter nematode movement, and some mediate repulsion of nematodes from the root (Chin et al., 2018; Sikder and Vestergård, 2020). The plant hormone ethylene is also implicated in regulating the attractiveness of the root to parasitic nematodes. Several studies found that plant mutants defective in ethylene signaling are less attractive to root knot nematodes, while ethylene overproduction reduces their attraction (*e.g.* Fudali et al., 2013; Čepulyte et al., 2018).

J2s enter the host root just behind the tip and travel intercellularly to the root tip, where they reverse direction and travel into the vascular cylinder of the root (Jones, 1981). Within 24 h, the nematode typically starts to initiate a feeding site by injecting a multitude of effectors into vascular parenchyma cells (Mejias et al., 2019). Often, several cells are targeted by one female (Bird, 1959). Some of the effectors play a role in controlling host defense responses (*e.g.* Sato et al., 2019), while others control the development of the feeding site (Davis et al., 2008; Viera and Gleason, 2019). The target cells undergo endoreduplication, which leads to the formation of multinucleate ‘giant cells’ (Goverse et al., 2000; Favery et al., 2002). The expansion of the giant cells inside the vascular cylinder is accompanied by multiple divisions of pericycle and cortical cells in the surrounding cell layers, which leads to the formation of a root gall, in which the female develops (Jones, 1981).

The formation of root galls shows some developmental similarities to the formation of root nodules in legumes (Mathesius, 2003; Bird, 2004). Nodulation is initiated by Nod factors produced by symbiotic bacteria called rhizobia. Nodule development in legumes starts with cell cycle activation and cell division in the pericycle and cortex of the host root (Xiao et al., 2014) and leads to the formation of differentiated nodules, in which rhizobia fix atmospheric nitrogen (Oldroyd, 2013). However, giant cell formation is unique to gall formation. Nodule formation has been studied in detail in several legumes, and many of the genes required for the infection of rhizobia and the development of nodules have been characterized (Roy et al., 2020). The symbiosis starts with the exudation of specific root flavonoids by the host, which activates the synthesis of nodulation (Nod) genes in compatible rhizobia (Peters et al., 1986). Nod gene activation results in the synthesis of Nod factors, which are perceived by specific receptors on the root surface, encoded by LysM receptor kinases (Radutoiu et al., 2003). In the model legume *Medicago truncatula*, these are encoded by *NFP1* and *2* (*NOD FACTOR PERCEPTION1/2*) (Amor et al., 2003; Arrighi et al., 2006). This activates a signaling cascade that involves calcium spiking and the activation of transcription factors inside the infected root hair that are necessary for successful nodulation. Essential genes for nodulation include the transcription factors *NIN* (*NODULE INCEPTION;* Vernié et al., 2015) as well as *NSP1* and *2* (*NODULATION SIGNALING PATHWAY1/2;* Kaló et al., 2005), which activate downstream genes, including the gene encoding the cytokinin receptor *CRE1* (Gonzalez-Rizzo et al., 2006; Plet et al., 2011). Mutation of *NFP1/2*, *NIN1* or *NFP1/2* completely abolishes nodulation, while mutation of *CRE1* significantly reduces nodule numbers, but does not completely prevent the formation of nodules (Murray et al., 2007; Plet et al., 2011). Infection and nodule number are further under negative control of ethylene signaling (Larrainzar et al., 2015; Guinel, 2015). The ethylene insensitive mutant *skl* (*sickle*; Penmetsa and Cook, 1997), which encodes the ethylene signaling protein EIN2 (Penmetsa et al., 2008), is characterized by both hyperinfection by rhizobia as well as hypernodulation. Interestingly, the *skl* mutant is also hyperinfected by other organisms including the pathogenic fungus *Rhizoctonia solani*, the oomycete pathogen *Phytophthora medicaginis* as well as symbiotic mycorrhizal fungi (Penmetsa et al., 2008). The likely explanation for those phenotypes is that ethylene plays an important role in plant defense (Broekgaarden et al., 2015).

Another plant hormone that interferes with nodule development is abscisic acid (ABA), which inhibits Nod factor signaling, including calcium spiking responses that occur following Nod factor perception, as well as the induction of several early nodulation genes (Ding et al., 2008). Nodule numbers, as well as lateral root numbers, are significantly reduced in the ABA-insensitive *latd/nip* (*lateral root organ defective/numerous infections and polyphenolics*) mutant of *M. truncatula* (Bright et al., 2005; Liang et al., 2007). However, it was also found that LATD/NIP encodes a nitrate transporter and thus the exact function of LATD/NIP in nodulation remains unclear (Yendrek et al., 2010). Nevertheless, the mutant shows generally defective root organ numbers and root apical meristem defects and is thus an interesting mutant to study during the formation of nematode galls, which also involve the initiation of cell divisions, similar to other root organs.

Both nodules and galls develop into nutrient sinks that consume host resources to some extent (*e.g.* Carneiro et al., 1999; Voisin et al., 2003). Legumes have evolved an autoregulation of nodulation mechanism that limits nodule numbers to levels adjusted to the nitrogen need of the plant. This mechanism involves the synthesis of small peptides of the CLE (CLAVATA3/ESR-RELATED) family following Nod factor perception and *NIN* activation (Ferguson et al., 2019). The CLE peptide CLE12 is further arabinosylated by RDN (ROOT DETERMINED NODULATION; Kassaw et al., 2017) and travels to the shoot, where it is perceived by a receptor kinase named SUNN (SUPER NUMERIC NODULES) in *M. truncatula* (Schnabel et al., 2005). This leads to the generation of one or more inhibitory signals travelling to the root to inhibit further nodule formation. These signals include changes in auxin transport in *M. truncatula* (van Noorden et al., 2006), altered cytokinin transport in *Lotus japonicus* (Sasaki et al., 2014) and shoot-to-root transport of a microRNA (miR2111) in *L. japonicus* (Tsikou et al., 2018).

So far, it is not clear whether genes involved in nodule development are also required for gall formation and *vice versa*. Some of the genes involved in gall formation are also activated during nodule formation, including genes playing a role in meristem activation (*PHAN* and *KNOX*), the early nodulin *ENOD40* and the cell cycle regulator *CCS52* (Koltai et al., 2001). The dividing cells of nodules and galls also show similarities in auxin responses and in the activation of flavonoids, which may play a role in the control of auxin transport or defense during the interactions (Hutangura et al., 1999; Mathesius, 2003). However, while flavonoids are essential for nodule development through their action on Nod gene activation and auxin transport (Wasson et al., 2006), flavonoid-deficient transgenics roots of *M. truncatula* still form galls, although somewhat smaller (Wasson et al., 2009). A transcriptome comparison of genes expressed in nematode-induced galls and the nodule zone II, which is the zone actively infected by rhizobia, showed a significant overlap in the expression of many genes in *M. truncatula* infected by its symbiont *Sinorhizobium meliloti* or the RKN *M. incognita* (Damiani et al., 2012). In the model legume *L. japonicus*, it was observed that nematodes cause similar root hair deformations, accompanied by cytoskeletal changes, as rhizobia, and it was hypothesized that nematodes make Nod factor-like molecular termed Nem-factors, although their existence has so far not been confirmed (Weerasinghe et al., 2005). In addition, the *L. japonicus* Nod factor perception mutants *nfr1* and *nfr5* showed reduced gall numbers after infection with *M. incognita*, suggesting a possible overlap in the signal transduction required for both nodules and root galls (Weerasinghe et al., 2005). In addition, the *L. japonicus* supernodulation mutant *har1* was found to form twice the number of galls, although not in an expanded root zone as typically found for nodulation (Lohar and Bird, 2003). However, so far little progress has been made in the elucidation of common gene necessary for both interactions.

Here, we examined a number of nodulation mutants of the model legume, *M. truncatula*, which are either defective in nodule initiation or nodule number control. We show that none of the genes analyzed that are essential for nodulation are necessary for gall or giant cell formation, although some mutations did affect the total number of galls and eggs produced by the female infecting nematodes. We also investigated the interaction of rhizobia with RKN on the same plants, as in the field, legumes are often co-infected by both organisms. We found that in wild type plants, rhizobia inhibited gall formation, but that this relationship was altered in some of the nodulation mutants.

## Methods

### Plant Preparation and Growth Conditions

Wild type (WT) *Medicago truncatula* Gaertn. cv. Jemalong A17 seeds were purchased from the South Australian Research and Development Institute (SARDI), Adelaide, Australia. Nodulation mutants, *nfp1-1, nsp2-2,* and *nin1* were received from Giles Oldroyd (Cambridge University)*, cre1* from Florian Frugier (Université Paris-Saclay)*, latd/nip* from Jeanne Harris (University of Vermont)*, skl* from Douglas Cook (University of California Davis), *and sunn4* from Julia Frugoli (Clemson University).

Seeds were lightly scarified using fine sand paper and surface-sterilized in 6% (w/v) sodium hypochlorite for 10 min. Seeds were then rinsed five times with sterile MilliQ water, spread on a 10% (w/v) water agar in a 15 cm diameter round plate (Corning, USA) and stratified in the dark at 4°C for two days. Germination was initiated by inverting and incubating the plates at 25°C for 16 h. Seedlings with ~0.5–1 cm radicle length were transferred onto round 15 cm diameter plates containing Fåhraeus medium (Fåhraeus, 1957) enriched with 1.5 mM potassium nitrate (hereafter called ‘enriched Fåhraeus medium’), which supported both nodulation and RKN infection. Seedlings of the *skl* mutant were transferred onto 245 mm × 245 mm square petri dishes (Corning, USA) containing enriched Fåhraeus medium because of their longer root length. The bottom half of the plates was covered with black paper and plates placed in a semi-vertical position in a plastic tray. Plants were maintained in a controlled temperature room at 25°C with 16 h light and 8 h dark cycle with 150 µE light intensity.

### *Meloidogyne javanica* Preparation

The *Meloidogyne javanica* population was isolated from field samples collected in Kiola, New South Wales, Australia. These were identified morphologically through perineal pattern observation and genetically based on PCR with primers specific to *M. javanica* (Zijlstra et al., 2000). Axenic cultures of *M. javanica* were amplified and maintained on *M. truncatula* A17 in enriched Fåhraeus medium. Egg masses were collected from infected *M. truncatula* A17 roots using a pair of forceps and surface-sterilized with 0.06% (v/v) sodium hypochlorite for 4 min with intermittent shaking. The eggs were centrifuged at 1,467 g for 5 min and were rinsed thoroughly with 1 ml sterile MilliQ water three times. This was followed by sterilization with a cocktail of 60 mg/µl penicillin, 250 mg/µl streptomycin, 20 mg/µl kanamycin, and 10 mg/µl amphotericin B in 1 ml solution for 4 h with 15 rpm vertical rotation. Thereafter, the eggs were centrifuged and the rinsing step was repeated. The solution containing the eggs was pipetted as 300 µl droplets on a sterile 8 cm diameter round petri dish (Corning, USA), and eggs were hatched by incubating at 25°C for one week. Hatched J2s were collected from the droplets and were counted in three aliquots of 5 µl. Ten to 15 J2s were inoculated per root (unless otherwise specified) by directly pipetting the J2s onto the root tip.

### Rhizobia Preparation

*Sinorhizobium meliloti* strain 1021 was grown overnight on Bergersen’s Modified Medium (BMM) (Rolfe et al., 1980) in the dark at 28°C. A colony of bacteria was transferred to a 15-ml sterile Falcon tube containing 9 ml of liquid BMM and incubated at 28°C overnight on an orbital shaker. The optical density of the resulting suspension was adjusted to 0.1 (at 600 nm) with sterile water before pipetting onto plant roots.

### Plant–Nematode Interaction Studies

J2s were inoculated on the root tips of one-week-old seedlings. At 35 days post inoculation (d.p.i.), the number of galls was counted using a stereomicroscope (Nikon SMZ745, Nikon, Japan). Five galls from each genotype were embedded in 3% (w/v) agarose and cross-sectioned at 110 µM thickness on a Vibratome 1000 plus (Vibratome Company, USA). Sections were viewed under a Leica DMLB microscope (Leica, Germany) under bright field illumination with a mounted CCD camera (RT Slider, USA). The remaining galls were used to harvest egg masses for nematode egg counts. The gelatinous matrix of egg masses was dissolved in 0.6% (w/v) sodium hypochlorite and processed as mentioned above. Eggs were counted one week after harvesting, and counts were based on three aliquots of 10 µl from the original suspension of 1 ml using a microscope (Leica Laborlux 11, Leica, Germany). Unhatched eggs and hatched J2s were counted for each aliquot to calculate total offspring and hatching rate (% of hatched J2s/total number of eggs).

### Plant–Nematode–Rhizobia Interaction Studies

A multi-factor experiment was designed to assess the phenotype of the selected *M. truncatula* nodulation mutants to *S. meliloti* and *M. javanica* infection. The two organisms were inoculated alone or in combination to establish whether these interact *via* the plant, potentially changing the phenotype.

Wildtype A17 and nodulation mutants growing on enriched Fåhraeus medium in petri dishes were each inoculated with 5 µl suspension of *S. meliloti* at OD = 0.1 (R treatment), 5 µl nematode suspension containing five *M. javanica* J2 (N treatment) or with both the rhizobia and the nematodes, inoculated immediately after one another (NR treatment). Inoculation was done by pipetting the suspensions directly onto the root tip. Each treatment was replicated 25 times.

After three weeks, numbers of galls and nodules of each replicate were recorded. Five plants of each treatment were further processed for visualization of nematodes in root tissue to ascertain whether genotypes differed in being able to be infected. For this, nematodes in root tissue were stained red through an adaptation of the Acid Fuchsin staining protocol (Byrd et al., 1983), adjusted for very fine roots. Briefly, whole roots were collected from the plates, transferred to 1.5 ml microtubes, and completely covered with a boiling solution of Acid Fuchsin stain. The stain solution was prepared by adding 10.5 ml of the stain stock solution (1.75 g Acid Fuchsin, 125 ml acetic acid, 375 ml distilled water) to 315 ml distilled water. The microtubes were left to cool overnight and roots were then rinsed in tap water to remove the stain and kept in glycerol:lactic acid (1:1). Nematodes inside roots were observed using a stereomicroscope, and their developmental stage was ascertained according to Triantaphyllou and Hirschmann (1960). Numbers of fully-developed females, or J2 through to J4 nematodes were recorded for each observed root.

### Statistical Analysis

Data were tested for normal distribution and homogeneity of variances prior to ANOVA. One-factor designs were analyzed by one-way ANOVA and pairwise comparisons were done by the *post-hoc* Tukey test. Assays with combined rhizobia and nematode inoculations were analyzed by two-way ANOVA, and significant differences caused by main effects or their interactions compared by the Bonferroni *post-hoc* test. All analyses were performed in GraphPad Prism 5.0 (GraphPad Software, San Diego, California).

## Results

### RKN Infection in *M. truncatula* Nodulation Mutants

**Figure 1:**
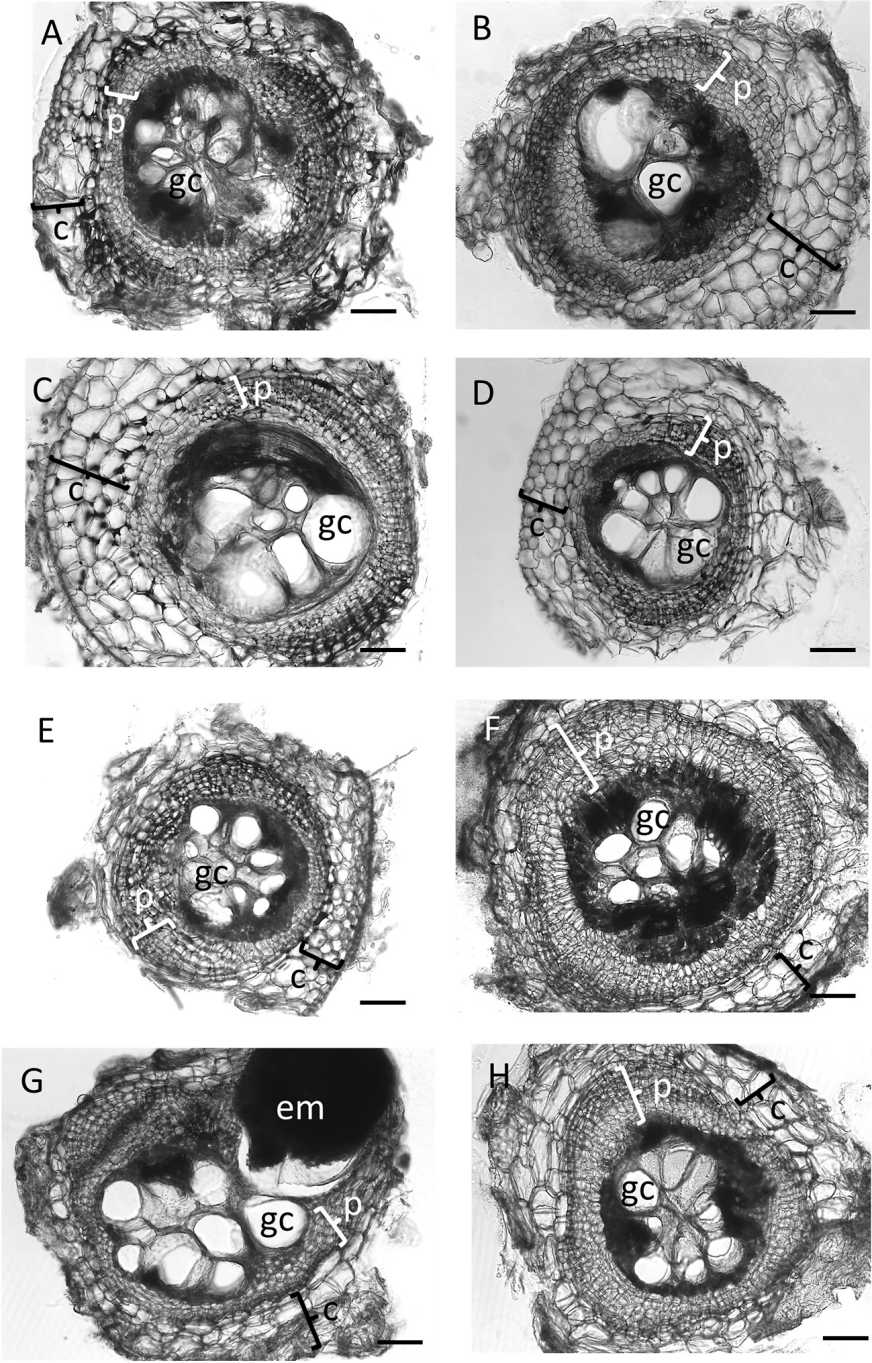
Cross sections of galls of wild-type *Medicago truncatula* A17 and nodulation mutants at 35 days post inoculation with *Meloidogyne javanica.* All photos were taken at the same magnification and the scale bar represents 200 µm. In total, five galls were sectioned from every genotype, and similar results were found for all five galls; thus these examples are representative of the replicates. **(A)** Gall section of *M. truncatula* A17, **(B)** *nfp1*, **(C)** *nin1*, **(D)** *nsp2-2,* **(E)** *cre1*, **(F)** *latd/nip*, **(G)** *skl* **(H)** *sunn4* mutants. Typical features of a gall are labeled as follows: c, cortex, occasionally showing divisions; em, egg mass; gc, giant cell (only one giant cell is labeled per section); p, pericycle, showing multiple layers following division.

**Figure 2:**
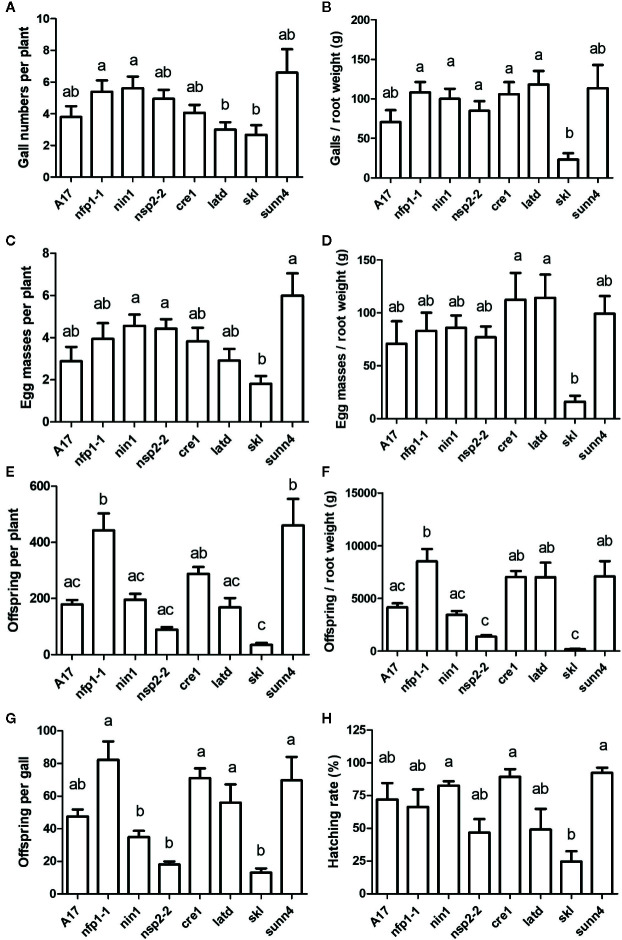
Nematode infection phenotypes of *Medicago truncatula* wild type and nodulation mutants. J2s of *Meloidogyne javanica* grown initially on WT plants were hatched and inoculated onto roots of different *M. truncatula* genotypes. All phenotypes were recorded at 35 days post inoculation. **(A)** Numbers of galls per plant. **(B)** Number of galls normalized against root weight. **(C)** Number of egg masses per plant. **(D)** Number of egg masses normalized against root weight. **(E)** Total offspring per plant (sum of unhatched eggs and hatched J2s after one week of incubating the harvested egg masses). **(F)** Total offspring normalized against root weight. **(G)** Total offspring per gall. **(H)** Hatching rate calculated as the numbed of hatched J2s/total number of eggs and J2s after one week of incubating the harvested egg masses. N = 17–25 plants for each genotype, with n = 5 for *sunn4*. Graphs show means and standard errors. Means with different letters differ significantly from each other with p < 0.05 (one-way ANOVA with Tukey’s post-test).

First, we tested the ability of a number of nodulation mutants to establish feeding sites (galls) following infection by *M. javanica*. All nodulation mutants, including the non-nodulating mutants *nfp1-1*, *nin1,* and *nsp2-2*, the low nodulation mutants *cre1* and *latd/nip*, the hypernodulating mutant *skl,* and the supernodulating mutant *sunn4* successfully formed galls (**Figures 1** and **2A**). Gall morphology was similar across the different mutants, and all genotypes supported the formation of giant cells and of pericycle and cortical cell divisions surrounding the giant cells (**Figure 1**). Despite their ability to successfully form galls, the genotypes differed quantitatively in their ability to host the RKNs through the formation of galls and the production of eggs (**Figure 2**). None of the non-nodulating mutants were compromised in their ability to form galls (**Figure 2A**). The lowest number of galls was formed in the hypernodulating *skl* mutant (**Figure 2A**), and this was even lower after normalizing gall numbers by root weight (**Figure 2B**).

The supernodulating mutant *sunn4* did not form significantly increased numbers of galls, suggesting that gall formation is not under autoregulation control in *M. truncatula*. To confirm that this result was not due to a limitation in the number of infecting J2, we conducted an additional experiment with WT and *sunn4* mutant plants, in which we increased the number of J2s to up to 50 nematodes per root. While both WT and *sunn4* mutants showed increased numbers of infecting nematodes and galls with increased numbers of inoculated J2s, the *sunn4* mutant still did not form more galls than the WT, in fact, gall numbers were lower than WT across inoculum levels (**Figure S1**). Gall numbers reached an average maximum of around five galls per root even with inoculation of 50 J2s, indicating that not all inoculated nematodes infect or form galls. Overall, gall numbers in the *sunn4* mutant roots varied somewhat between experiments relative to WT roots, but were never significantly higher than in WT roots in any of the experiments.

Galls of all genotypes also supported the formation of egg masses. Egg mass numbers per plant followed a similar pattern to gall number per plant, as most galls formed one egg mass. Again, the *skl* mutant showed significantly reduced eggs masses per root and egg masses per root weight compared to other genotypes (**Figures 2C, D**). Because egg masses contain variable number of eggs, we quantified the number of offspring (total of eggs and hatched J2s at one week after harvesting egg masses) and found significant differences between genotypes, with the *skl* mutant again showing the least number of offspring, while the *nfp1-1* and the *sunn4* mutants produced the highest numbers of offspring per plant and the *nfp1-1*, cre1, *latd/nip* and *sunn4* mutants supporting the highest number of offspring per root weight (**Figures 2E, F**). Similar figures were recorded when considering eggs produced per gall (**Figure 2G**). The percentage of eggs that resulted in hatched J2s were again the lowest for the *skl* mutant (**Figure 2H**).

**Figure 3:**
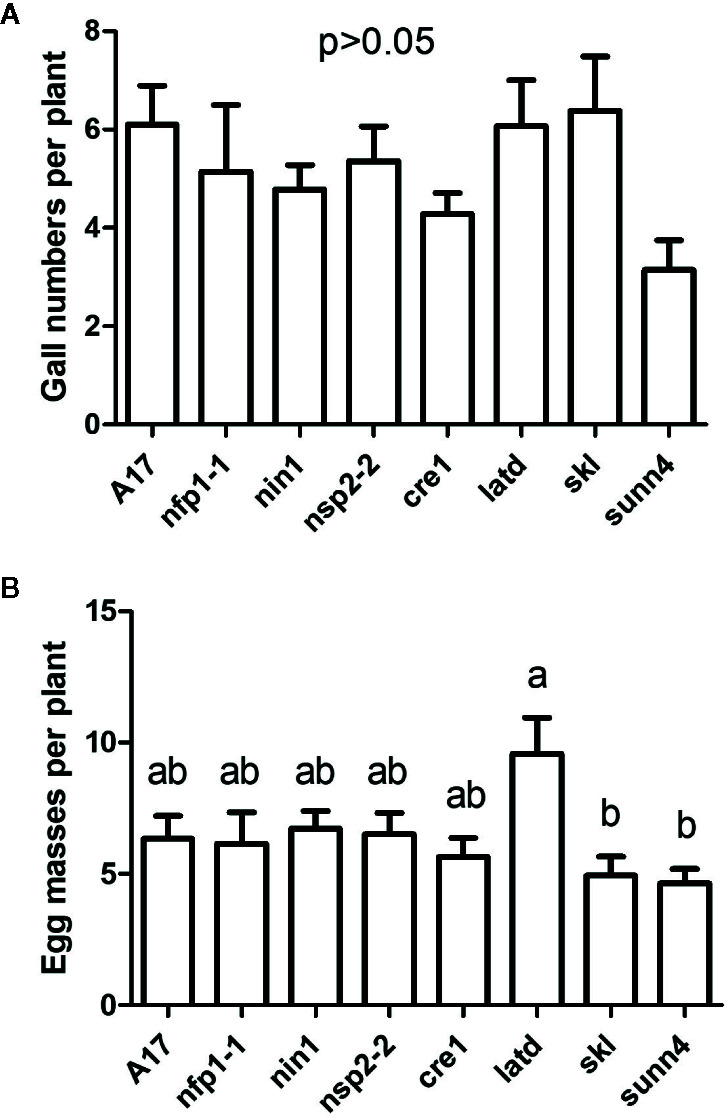
Nematode infection phenotypes of *Medicago truncatula* wild type roots infected with J2s hatched from eggs harvested from different nodulation mutants. **(A)** Number of galls per plant. No significant differences were found between egg origin from different genotypes. **(B)** Number of egg masses per plant. N = 14–21 plants for each genotype. Graphs show means and standard errors. Means with different letters differ significantly from each other with p < 0.05 (one-way ANOVA with Tukey’s post-test).

To test whether the hatched J2s were infective, we inoculated the hatched J2s originating from each genotype onto a new generation of WT roots. There were no significant differences between egg origin from different genotypes in the number of galls per plant formed in the subsequent generation (**Figure 3A**). The number of egg masses per plant was higher when J2s originated from the *latd/nip* mutant, but this was only significant in comparison with the *skl* and *sunn4* mutants (**Figure 3B**). Overall results confirmed that eggs produced by nematodes on all genotypes were viable.

### Co-Inoculation of Roots With *M. javanica* and *S. meliloti*

**Figure 4:**
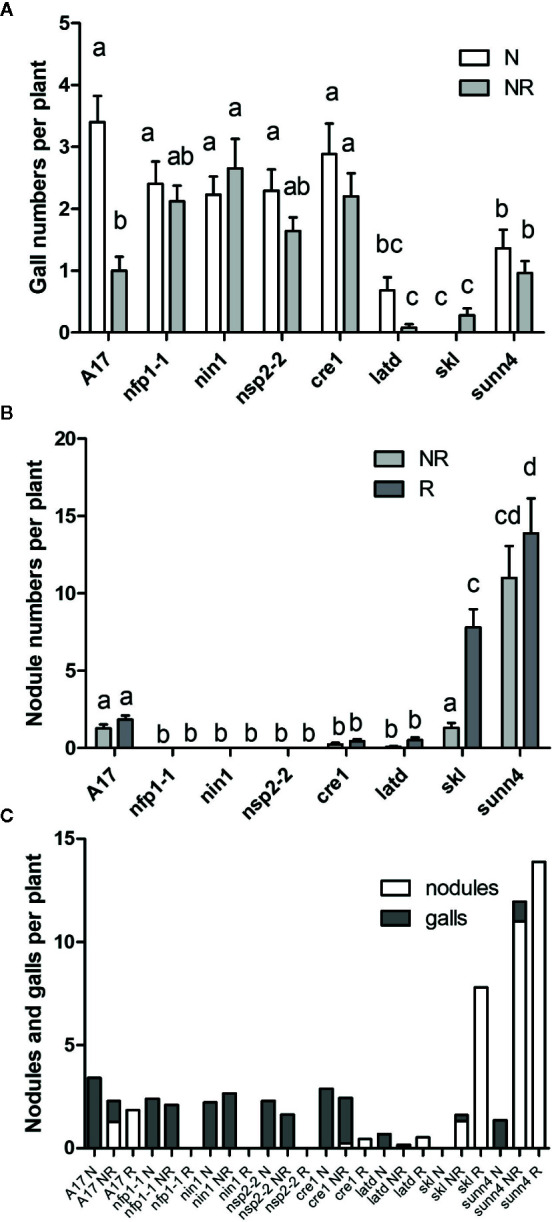
Gall and nodule formation in *M. truncatula* WT and nodulation mutants. Plants were either infected by nematodes only (N), by rhizobia only (R), or by rhizobia and nematodes at the same time (NR). Phenotypes were recorded 3 weeks after inoculation. **(A)** Number of galls per plant. **(B)** Number of nodules per plant. **(C)** Total number of galls and nodules. N = 25 plants for each genotype. Graphs show means and standard errors. Means with different letters differ significantly from each other with p < 0.05 (two-way ANOVA with Bonferroni post-test).

**Figure 5:**
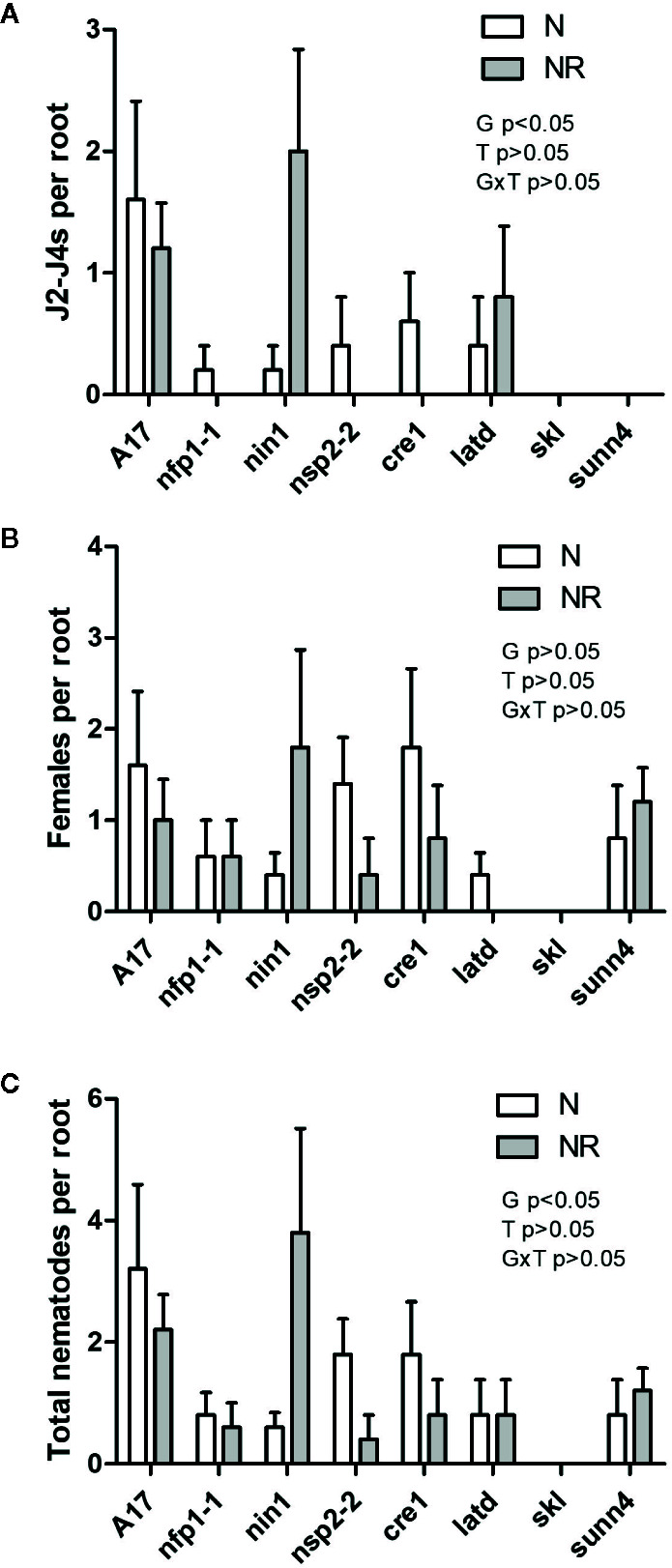
Quantification of nematodes localized inside *M. truncatula* roots. Roots were stained with Acid Fuchsin and both juvenile and adult stage *M. javanica* were counted in each root (examples of these stages are shown in **Figure S3**). **(A)** Number of J2 to J4 stage nematodes. **(B)** Number of adult stage nematodes. **(C)** Total number of juveniles and adults per root. N = 5. Graphs show means and standard errors. Significant effects of genotype (G), treatment (T), or their interaction (G × T) are shown in each panel but as interactions were not significant, no pairwise comparisons could be made (two-way ANOVA with Bonferroni post-test).

As legumes are typically infected by nodule-forming rhizobia in the field, we tested the interaction of nematodes and rhizobia on the same root system (**Figure S2**). Concurrently, controls with inoculation of roots with rhizobia only or nematodes only were set up. We harvested these plants at three weeks post inoculation, which was late enough to ensure galls and nodules were clearly visible, but early enough to avoid large nutritional effects due to nitrogen fixation, which would be low at this early stage. Co-inoculation of wild type plants with RKN and rhizobia together significantly reduced the number of galls compared to RKN-only infected roots (**Figure 4A**). A much smaller, and not statistically significant drop was recorded in the *nfp1*, *nsp2-2*, *cre1*, *latd/nip*, *skl* and *sunn4* mutants (**Figure 4A**). We also examined the total number of RKN that had infected the root because not all nematodes that infect also succeed in causing gall formation (**Figure S3**). We did not find any evidence that RKNs were present in roots with low gall formation, *e.g.* in the *skl* mutant (**Figure 5**), suggesting that reduced gall formation in the *skl* mutant is due to reduced numbers of nematodes entering the root, or remaining in the root after initial infection without being able to initiate a gall.

Co-inoculation of wild type plants with RKN and rhizobia together did not significantly reduce the numbers of nodules in WT plants compared to rhizobia-only inoculated roots (**Figure 4B**). Nodule numbers were very low and did not significantly change in the *cre1* or *latd/nip* mutants in co-inoculated plants. In contrast, in the hypernodulating *skl* mutant, co-inoculation with nematodes reduced the numbers of nodules significantly compared to rhizobia-only inoculated roots, with a lesser, but not statistically significant, drop in nodule numbers in the *sunn4* mutant (**Figure 4B**). The total number of galls and nodules in the different genotypes is shown in **Figure 4C**. This figure shows that there was no clear relationship between the ability of genotypes to host either nodules or galls.

## Discussion

Our aim was to characterize the interaction of RKN with a number of *M. truncatula* mutants defective in different stages of nodule formation. Firstly, we found that all nodulation mutants were able to form galls with well-formed giant cells, and that females in galls formed on all the nodulation mutants produced viable eggs. This shows that none of these nodulation genes are essential for successful feeding site formation by RKN. Our results did not support previous findings in *L. japonicus* showing that the non-nodulation mutants *nfr1* and *nfr5*, defective in Nod factor perception, showed reduced numbers of galls and mature females in *L. japonicus* roots infected with *M. incognita* (Weerasinghe et al., 2005). It is possible that the different species of plants and nematode species or the growth conditions used could explain this difference, but both studies agreed with the observation that Nod factor perception and early signal transduction genes were not absolutely required for gall formation.

As some of the early nodulation genes are required for rhizobial infection, and the infection mechanisms of rhizobia (*via* infection threads through root hairs; Murray, 2011) and RKN (intercellularly between cells; Jones, 1981) are quite different, the finding that early nodulation genes are not essential for nematode infection might not be surprising. On the other hand, early nodulation genes also control immune responses, and quantitative differences in infection of nodulation mutants with pathogens have been observed previously. For example, the *nfp1* mutant is more susceptible to infection by the oomycete *Phytophthora palmivora* (Rey et al., 2015), the oomycete *Aphanomyces euteiches* and the pathogenic fungus *Colletotrichum trifolii* (Rey et al., 2013). In contrast, the *latd* mutant was less susceptible to *P. palmivora*, while the *nin1* and *nsp2* mutants were similarly infected as the WT (Rey et al., 2015). The NFP receptor perceives *N*‐acetylglucosamine derivatives like Nod factors. The presence of related signals (Nem factors) by RKN has been hypothesized because of the presence of *NodC* genes in RKN genomes and because RKNs have been observed to cause root hair curling, similar to the effect of rhizobial Nod factors (Weerasinghe et al., 2005); however Nem factors have so far not been identified.

While Nod factor perception is necessary for rhizobial infection and nodule development, legumes can activate the nodule development program in the absence of rhizobia and without Nod factor perception through spontaneous nodule formation, for example in alfalfa (Joshi et al., 1991). Ectopic overexpression of *NIN* (Vernié et al., 2015) or activation of the cytokinin signaling pathway (Tirichine et al., 2007; Heckmann et al., 2011; Gauthier-Coles et al., 2019) also induces nodule initiation by activating cortical cell divisions, suggesting that the nodule development program does not require Nod factor perception but does require cytokinin signaling. If gall formation requires similar signaling, the formation of pericycle and cortical cell divisions as part of the gall development program would therefore have been expected to require Nod factor signaling through NIN and CRE1, as well as NSP2 (which acts downstream of CRE1), but not NFP1 (Madsen et al., 2010). It is possible that nematodes directly target cytokinin signaling in the root to establish feeding sites, as RKN can synthesize cytokinins (de Meutter et al., 2003), although this might not be sufficient for maintaining sustained cell divisions.

While early nodulation genes were not essential, the nodulation mutants did differ quantitatively in their ability to host galls and eggs produced by infecting females. The one mutant that showed repeatable and significant differences compared to other genotypes was the *skl* mutant, which formed significantly fewer galls, hosted fewer nematodes inside roots, with females producing fewer eggs with a lower hatching rate. Our observations in *M. truncatula* were consistent across two different experiments and were found in the absence and presence of rhizobia. This result was surprising because the *skl* mutant is hyperinfected by both rhizobia as well as symbiotic mycorrhizal fungi and root pathogens, including *Phytophthora* and *Rhizoctonia* (Penmetsa et al., 2008), and this is thought to be a result of reduced defense responses in ethylene-insensitive plants (Broekgaarden et al., 2015; Berrabah et al., 2018). When RKNs infect host roots, host defense responses are initially observed, although there is also evidence that the infective females inject effectors into target cells that prevent defense responses at the stage of giant cell initiation (Goto et al., 2013; Ji et al., 2013; Goverse and Smant, 2014). In tomato roots, a comparative transcriptomics study showed that ethylene-related responses were more strongly induced in resistant tomato genotypes in response to *M. incognita* than in a susceptible genotype, suggesting a role for ethylene in RKN defense (Shukla et al., 2018).

Ethylene is also required for induction of some of the systemic plant defense responses activated by jasmonic acid, and application of the ethylene releasing compound ethephon to leaves of rice was shown to reduce infections of rice roots with *M. graminicola* (Nahar et al., 2011). To what extent lack of ethylene signaling regulates the local defense responses to nematodes is less well studied, but our results agree with observations that *M. javanica* induced ethylene production in tomato roots and more strongly so in susceptible cultivars, and that application of ethylene inhibitors reduced infection, suggesting that ethylene is required for successful RKN infection (Glazer et al., 1983; Glazer et al., 1985). Ethylene has also been implicated at an earlier stage of the interaction by affecting chemotaxis of nematodes towards the root. Ethylene-insensitive mutants of Arabidopsis and tomato were previously shown to attract more nematodes (Fudali et al., 2013). However, we suggest that this is unlikely to be a factor influencing our results because nematodes were inoculated directly at the root tip and thus did not have to move towards the root. In addition, if the *SKL* mutation acted similarly to other ethylene signaling defects in other species, the *skl* mutant should have attracted more, rather than fewer RKNs. In future work, it would be interesting to compare RKN infections in a number of ethylene-related mutants that vary in the parts of the ethylene pathway that is defective.

While lack of ethylene signaling was expected to enhance nematode infection, we expected that lack of cytokinin signaling would reduce gall formation. Cytokinin is critical for the control of cell division and the cytokinin response gene *ARR5* was induced during the early stages of giant cell formation and in surrounding dividing cells of *L. japonicus* and tomato roots infected by RKN (Lohar et al., 2004). In Arabidopsis, cytokinin synthesis genes and cytokinin receptors were also activated during gall formation (Dowd et al., 2017) and the Arabidopsis cytokinin perception (double) mutants *ahk2/3, 2/4* and *3/4* were characterized by reduced numbers of galls (Dowd et al., 2017). The *M. truncatula CRE1* gene is a homolog of *AtAHK4* (Gonzalez-Rizzo et al., 2006). It is likely that the normal gall formation in the *cre1* mutant was observed because other Medicago cytokinin receptors were able to compensate for the lack of *CRE1*. Partial redundancy of cytokinin receptors has also been observed during nodulation (Held et al., 2014).

The results observed in the *latd/nip* mutant varied slightly between experiments. While the first experiment did not show significant reductions of gall numbers in the *latd/nip* mutant (**Figure 2**), reduced galls and infecting RKNs were observed in the second experiment (**Figures 4** and **5**). It is possible that this was due to different harvest times and number of nematodes used for infection. The *latd/nip* mutation leads to ABA insensitivity, although this is likely not the only reason for its meristem defect because LATD/NIP encodes a nitrate transporter. In rice and tomato, addition of ABA increased root susceptibility to RKN, but the detailed role of ABA in plant-RKN interactions will need to be investigated in more detail in the future.

Despite its supernodulation phenotype, the autoregulation mutant *sunn4* did not show increased numbers of galls, while increased nodule numbers in the *sunn4* mutant were observed under the same conditions, as expected (Schnabel et al., 2005). This suggests that signals that trigger autoregulation of nodulation are not activated by *M. javanica* and agrees with the lack of requirement for Nod factor signaling genes during RKN infection. In *M. truncatula*, Nod factor signaling through NIN, NSP2 and CRE1 is required for activation of autoregulation (Mortier et al., 2010; Mortier et al., 2012), but the *nin1, nsp2-2* and *cre1* mutants were not defective in gall formation in our study, indicating that this signaling pathway is not activated by RKN. Our finding was in contrast to the observation in *L. japonicus* that the supernodulating mutant *har1*, defective in the ortholog of SUNN, formed higher number of galls (Lohar and Bird, 2003). It is possible that the different plant species behave somewhat differently, especially because the *nfp* mutants of *L. japonicus* also showed reduced numbers of galls (Lohar and Bird, 2003).

A further question of our study was to explore the interaction of rhizobia and nematodes in the same root system. A previous study found a correlation between the ability of different ecotypes of *M. truncatula* to form galls and nodules (Wood et al., 2018). This suggests common genetic regulation of both interactions, although the molecular basis for this is not understood. However, both organisms also affect each other indirectly through their effects on the host or each other. Rhizobia as well as a number of other soil bacteria often contribute to preventing plant infection by parasitic nematodes, and this can involve changes in plant (systemic) defenses, root exudates that affect both organisms, while some bacteria and fungi can also directly kill or trap nematodes (Topalović et al., 2020). Thus, we were interested to see what effect rhizobia had on nematode gall formation and if this was controlled by any of the selected nodulation genes.

We found that the number of galls formed were influenced by concurrent inoculation with rhizobia, however, this was only significant in WT plants. The observed drop in gall numbers in rhizobia-coinoculated roots could be due to changes in defense, exudation or other responses in the root induced by rhizobia (Zamioudis and Pietersé, 2012; Topalović et al., 2020), but it could also be due to resource competition of the established organs, as both nodules and galls act as nutrient sinks in the root (Carneiro et al., 1999; Voisin et al., 2003). While resource competition between nodules and galls could account for the drop in galls in WT roots, this drop was not seen in other genotypes that nodulated even better, e.g. the *skl* and *sunn4* mutants. The second explanation is the possible alteration of defense-related responses in rhizobia-inoculated roots. However, this is difficult to explain in view of the inconsistent responses in the different mutants, *i.e.* both hyper- and non-nodulating mutants lacked a drop in gall numbers in the presence of rhizobia. Our experiments were additionally complicated by the fact that plants grown on agar plates typically do not nodulate as well as soil-grown plants, and this involves the induction of ethylene in the plate (Smith and Long, 1998), which is likely to affect gall numbers as well. Therefore, the interpretations of our results need to be viewed with some caution and will require further studies in soil-grown plants. Moreover, the nodulation mutants, in particular *cre1*, *latd/nip*, *skl* and *sunn4* have other root architecture phenotypes, which could influence the results indirectly (Penmetsa and Cook, 1997; Bright et al., 2005; Schnabel et al., 2005; Desbrosses and Stougaard, 2011; Plet et al., 2011). For example, differences in speed of root growth, number of lateral roots (providing entry points for nematodes) and differential C allocation to roots, galls and nodules could all indirectly influence the RKN infection phenotypes analyzed here. In addition, the interaction between gall and nodule numbers could be influenced by the differences in the size of the root system of different mutants. For example, the root system of supernodulation mutants is typically much smaller than in WT plants, and this changes with nodulation status (*e.g.* Wopereis et al., 2000; Schnabel et al., 2005; Goh et al., 2019). Not all of these phenotypes were captured in our experiments. Thus, a systematic study of changes to the whole root architecture in these mutants in the presence and absence of different parasites and symbionts would be fruitful, especially in soil and field-grown plants.

Co-inoculation of RKNs and rhizobia also resulted in changes to nodule numbers, although the only significant reductions in nodule numbers were seen in the hyper-nodulating *skl* mutant. This suggests that, even though RKN induced very low numbers of galls in the *skl* mutant, the RKN must have induced responses in the plants that interfered with nodulation. The *skl* mutant is thought to generally show reduced ethylene-mediated defense responses (Penmetsa and Cook, 1997; Penmetsa et al., 2008), but it is possible that this is compensated for by activation of defense responses that are not mediated by ethylene, and this would be interesting to investigate in the future. Resource competition between galls and nodules is also an unlikely explanation in this experiment because we did not observe a significant reduction in nodule numbers in the *sunn4* mutant, which would have had a strong carbon sink due to its high nodule numbers.

A drop in nodule numbers in nematode-infected root system has also been observed in other legumes (e.g. Khan et al., 2018). However, results are likely to be strongly influenced by the age and nutritional status of the plant, the time of harvest and the age of the galls and nodules. Additional complex interactions could be due to the contribution of fixed nitrogen from nodules, which we tried to minimize here by phenotyping the plants as early as possible. Future experiments could try to unravel these complex interactions in plants grown under a number of different conditions.

In summary, we found that none of the examined early signaling genes required for nodulation were essential for successful RKN parasitism. The autoregulation of nodulation gene *sunn4* was similarly not involved in controlling the numbers of galls in *M. truncatula*. We found evidence for a positive role of ethylene signaling in successful gall formation and egg production, in contrast to its negative role in nodulation, mycorrhization and other pathogen interactions. We found a protective effect of rhizobia on RKN parasitism although the mechanism of this protection remains unclear. A negative effect of RKN on nodule numbers was also observed, but only in the *skl* mutant, suggesting a role for ethylene signaling in the interaction of rhizobia and RKN in the root. The molecular mechanism for the action of ethylene signaling in RKN infection and feeding site formation will be an important goal in the future.

## Data Availability Statement

All datasets presented in this study are included in the article/**Supplementary Material**.

## Author Contributions

SRC and SC carried out the experiments. All authors contributed to the conception of the study and analysis of data. UM wrote the manuscript with input from all authors.

## Funding

SRC was funded by a post-doc grant (SFRH/BPD/26496/2006) supported by the Portuguese Foundation for Science and Technology (FCT). We also thank the Australian Research Council and the Grains Research Development Corporation for funding through grant # IC170100005.

## Conflict of Interest

The authors declare that the research was conducted in the absence of any commercial or financial relationships that could be construed as a potential conflict of interest.
