# Supplementary material for: Infection of Medicago truncatula by the Root-Knot Nematode Meloidogyne javanica Does Not Require Early Nodulation Genes
Source: Front Plant Sci. 2020 Jul 9;11:1050. doi: 10.3389/fpls.2020.01050 (PMC7363973; doi:10.3389/fpls.2020.01050)
Supplement: Supplementary file 1 [file DataSheet_1.docx]

Supplementary Material


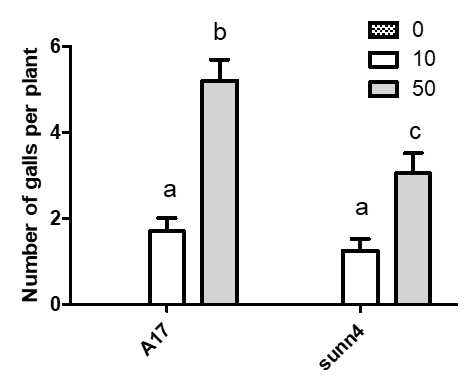


**Supplementary Figure 1.** Number of galls per plant in *Medicago truncatula* A17 and *sunn4* mutant roots inoculated with different numbers of *Meloidogyne javanica* J2s. Galls were counted after inoculation of roots with 0, 10 or 50 J2s per root and counted 21 days after inoculation. Error bars indicate standard error of the mean and letters indicate significant differences between treatments (p<0.01) following two-way ANOVA.


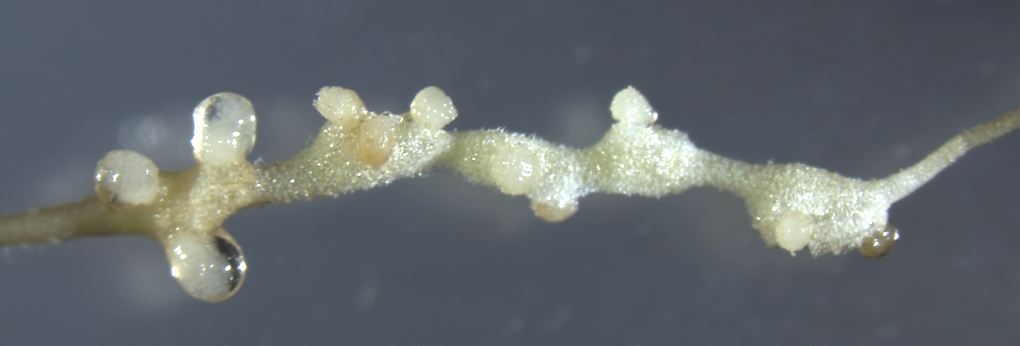

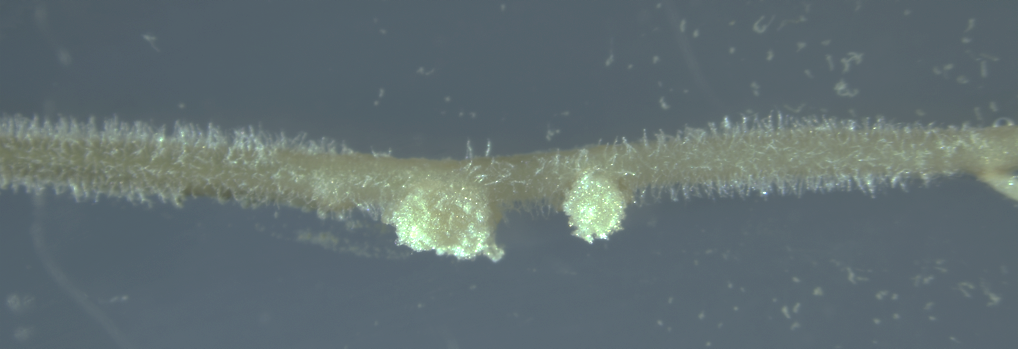


em

n

g

A

B

**Supplementary Figure 2.** Examples of galls and nodules on *Medicago truncatula* roots. Roots in (A) were infected with *Meloidogyne javanica* and are shown at 35 days post infection. *M. javanica* have caused the formation of root galls (g), many of which show the development of egg masses (em; also indicated with arrows). Roots in (B) were infected with *Sinorhizobium meliloti*, and are shown at 3 weeks post inoculation when small nodules (n) have formed. Magnification bar = 0.5 mm in (A) and 1 mm in (B).


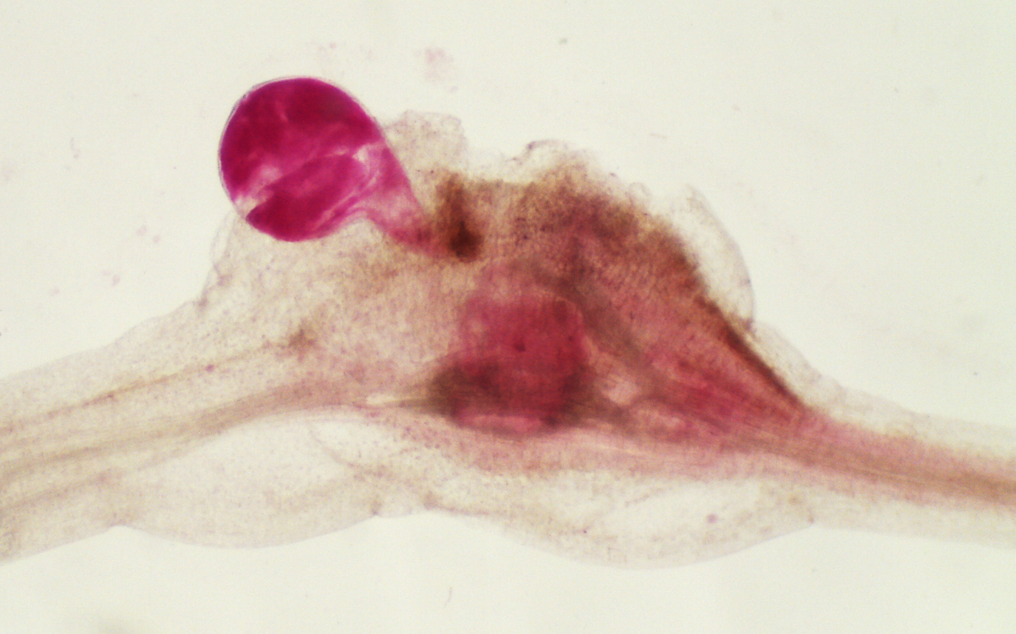

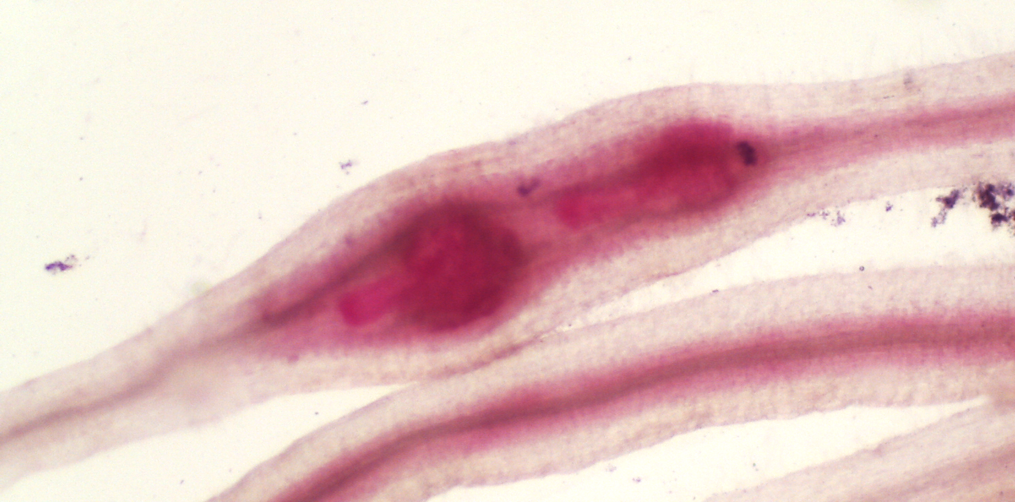

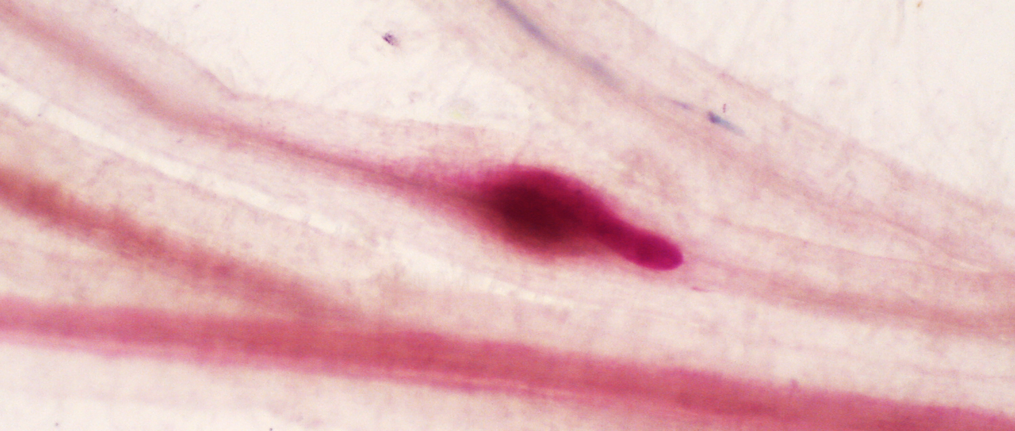


A

C

B

**Supplementary Figure 3.** Examples of *M. javanica* located inside *M. truncatula* roots. Panels (A) and (B) show juvenile stages while panel (C) shows an example of an adult female inside a gall. Nematodes were stained with Acid Fuchsin and are visible in bright pink (arrows). Magnification bar represents 1 mm.
